# Accumulation of prohibitin is a common cellular response to different stressing stimuli and protects melanoma cells from ER stress and chemotherapy-induced cell death

**DOI:** 10.18632/oncotarget.17810

**Published:** 2017-05-11

**Authors:** Tharcisio Citrangulo Tortelli, Lyris Martins Franco de Godoy, Gustavo Antonio de Souza, Diego Bonatto, Andreia Hanada Otake, Renata de Freitas Saito, Jose Cesar Rosa, Lewis Joel Greene, Roger Chammas

**Affiliations:** ^1^ Centro de Investigação Translacional em Oncologia (LIM24), Departamento de Radiologia e Oncologia, Faculdade de Medicina da Universidade de São Paulo and Instituto do Câncer do Estado de São Paulo, São Paulo, SP, Brazil; ^2^ Departamento de Biologia Celular, Molecular e Bioagentes Patogênicos, Faculdade de Medicina de Ribeirão Preto, Universidade de São Paulo, Ribeirão Preto, SP, Brazil; ^3^ Centro de Biotecnologia, Departamento de Biologia Molecular e Biotecnologia, Universidade Federal do Rio Grande do Sul, Porto Alegre, RS, Brazil; ^4^ Instituto Carlos Chagas, Fiocruz Paraná, PR, Brazil; ^5^ Instituto do Cérebro, Universidade Federal do Rio Grande do Norte, Natal, RN, Brazil; ^6^ Centro de Terapia Celular, Fundação Hemocentro de Ribeirão Preto, Ribeirão Preto, SP, Brazil

**Keywords:** melanoma, prohibitin, mitochondria, cisplatin, tunicamycin

## Abstract

Melanoma is responsible for most deaths among skin cancers and conventional and palliative care chemotherapy are limited due to the development of chemoresistance. We used proteomic analysis to identify cellular responses that lead to chemoresistance of human melanoma cell lines to cisplatin. A systems approach to the proteomic data indicated the participation of specific cellular processes such as oxidative phosphorylation, mitochondrial organization and homeostasis, as well as the unfolded protein response (UPR) to be required for the survival of cells treated with cisplatin. Prohibitin (PHB) was among the proteins consistently accumulated, interacting with the functional clusters associated with resistance to cisplatin. We showed PHB accumulated at different levels in melanoma cell lines under stressing stimuli, such as (i) treatment with temozolomide (TMZ), dacarbazine (DTIC) and cisplatin; (ii) serum deprivation; (iii) tunicamycin, an UPR inducer. Prohibitin accumulated in the mitochondria of melanoma cells after cisplatin and tunicamycin treatment and its *de novo* accumulation led to chemoresistance melanoma cell lines. In contrast, PHB knock-down sensitized melanoma cells to cisplatin and tunicamycin treatment. We conclude that PHB participates in the survival of cells exposed to different stress stimuli, and can therefore serve as a target for the sensitization of melanoma cells to chemotherapy.

## INTRODUCTION

Although it has a low prevalence, the incidence of melanoma is increasing worldwide. Manageable when diagnosed in the early phases with local surgery and complete removal of the tumor, advanced metastatic melanomas pose a challenge to treatment. Enrollment of advanced melanoma patients in clinical trials, such as those with Raf inhibitors like Vemurafenib [1] or blockers of immune checkpoints (e.g., Ipilimumab [2] and Nivolumab [3]) is considered to be the standard of care in developed countries since the results of treatment with conventional chemotherapeutic agents are considered to be poor. In underdeveloped countries, melanoma patients have no access to clinical trials of the newest drugs available, so that new approaches using less expensive strategies are still necessary. Clinical protocols for palliative care of melanoma patients in these countries include the use of cisplatin (CDDP) and dacarbazine (DTIC), which is metabolized into 5-[3-methyl-triazen-1-yl]-imidazole-4-carboxamide (MTIC) [4, 5]. Understanding why melanoma cells are resistant to conventional therapy and devising ways to increase melanoma sensitivity to chemotherapeutic agents may impact melanoma patient management in a cost-effective manner.

We have evaluated the development of resistance to cisplatin by human melanoma cells using a proteomic approach in order to identify putative targets for intervention. Cisplatin forms adducts with DNA and initiates a cell death program or a DNA repair system [6]. Several mechanisms protect melanoma cells from cisplatin-induced cell death, either (i) by activating DNA-repair systems such as the p53-dependent upregulation of the nucleotide excision repair (NER) genes Xeroderma pigmentosum complementation group C (XPC) and damaged DNA-binding protein 2 (DDB2) [7] or (ii) by pH-dependent sequestration and extrusion of cisplatin through exosomes [8]. Also, survival pathways such as the induction of mitogen-activated protein kinase (MAPK) can be activated, and this mechanism also leads to increased DNA repair [9]. Cisplatin can also induce oxidative stress in mitochondria by increasing the production of reactive oxygen species (ROS) [10], for example by uncoupling the respiratory chain [11]. The increase of ROS in melanoma cells can trigger several adaptive mechanisms that overcome ROS damage and cell death, including stabilization of hypoxia-inducible factor 1 (Hif-1α), which in turn protects the tumor from ROS-induced cell death [12]. The pattern of protein accumulation indicates that adaptive mechanisms include the unfolded protein response (UPR) pathway and accumulation of the mitochondrial chaperone prohibitin (PHB).

Here, using a proteomic approach, we showed that PHB was accumulated upon cisplatin treatment. Prohibitin accumulation was also observed in response to other stress stimuli, such as the chemotherapeutic agents DTIC and temozolomide (TMZ), the unfolded protein response inducer tunicamycin, and nutrient starvation. Since PHB knock-down sensitized melanoma cells which are sensitive to stress stimuli, PHB accumulation appears to be a targetable arm of a prosurvival cellular response.

## RESULTS

### Proteomic analysis of a cisplatin-treated melanoma cell line

LB373 cells were treated for 8, 16 and 24 h with 25 μM of cisplatin (EC50, Supplementary Figure 1). After 24h, cell death was evaluated by reduction of mitochondrial potential, phosphatidylserine exposure, as well as DNA fragmentation (Supplementary Figure 2).

An average of 500 ± 25 spots was detected for each reaction time after IEF-SDS-PAGE separation of extracts of the melanoma cells (Supplementary Figure 3). The number of spots in each gel was determined through the analysis program (ImageMaster-2D software). Triplicates of each treatment time were made, and some spots were not detected in all replicates, or at all treatment times. The number of spots per gel of 500 ± 25 indicates that the mean was 500 and ± 25 was the standard deviation. Comparison of the intensity of protein spots among the gels from cells untreated (time 0) and treated with cisplatin for 8, 16 and 24 h indicated that 111 “spots” showed quantitative variation of more than a two-fold increase or decrease for at least one incubation time (exemplified in Supplementary Figure 3B), indicating their regulation upon cisplatin treatment. Of these, 75 increased, 29 decreased, and 9 presented a variable response to cisplatin. The 34 regulated proteins identified by mass spectrometry, which comprise 62 isoelectric forms, are listed in Table 1.

**Table 1:** Time course of differential protein accumulation by LB373 melanoma cells after treatment with cisplatin

Identification	Spot	Relative Spot Intensity (h)		
		8	16	24
60 kDa Heat Shock Protein (Hsp60)	48	1.2	1.3	3.3
	50	3.2	3.5	n.d.
	52	3.1	2.6	1.8
	53	1.7	1.2	6.8
	67	0.1	0.5	0.1
78 kDa glucose-regulated precursor (GRP78)	39	2.0	1.4	4.6
	156	0.4	1.4	4.0
	317	2.0	2.4	5.8
	361	0.6	0.9	2.0
Actin, cytoplasmic 1 (Beta-actin)	146	3.5	6.0	4.1
	147	2.9	4.3	3.0
	150	3.6	3.7	6.8
	152	3.7	3.3	5.0
Alpha enolase	211	1.9	2.0	1.5
ATP Synthase beta chain, mitochondrial precursor	90	2.0	2.9	1.3
	98	0.6	0.7	12.0
ATP Synthase D Chain, mitochondrial	441	0.9	1.3	1.8
Calreticulin Precursor	120	0.9	1.4	0.3
	479	0.4	0.4	0.8
Cathepsin D precursor	328	0.7	2.2	1.9
	329	0.5	1.1	1.9
Endoplasmic reticulum protein ERp29 precursor	359	2.5	2.8	2.7
Endoplasmin precursor	45	n.d.	n.d.	8.2
Eukaryotic translation initiation facotr 3 subunit 2	214	1.1	2.0	2.0
	220	0.7	0.5	1.1
F-actin capping protein alpha-1 subunit	250	1.2	2.7	2.3
Galectin-1	514	0.8	1.0	1.9
	517	0.7	1.3	2.4
Glutathione S-transferase P	412	1.7	1.9	2.4
Heat-Shock protein beta-1	370	2.7	2.4	0.5
	375	2.2	4.6	3.3
Heat shock 70 kDa protein 1 (HSP70.1)	183	1.8	2.8	1.7
Inorganic pyrophosphatase	279	2.7	2.9	n.d.
Lamin A/C	376	0.9	3.9	4.4
Myosin light chain alkali, non-muscle isoform	500	1.4	2.4	2.9
Nucleophosmin	221	0.3	4.1	n.d
	222	1.5	3.2	n.d
	474	1.8	1.4	1.3
Nucleoside diphosphate kinase A	498	0.4	0.3	0.7
Peroxiredoxin 4	371	0.5	1.2	2.0
Peroxiredoxin 6	383	2.0	1.1	1.4
Phosphoglycerate kinase 1	396	0.8	0.5	1.1
Prohibitin	339	1.4	1.6	2.0
Protein disulfide isomerase A3 precursor	22	2.6	3.1	1.1
	55	3.4	3.0	4.5
	57	3.5	3.7	2.6
	347	1.1	1.1	2.8
Rho GDP-dissociation inhibitor 1	384	0.9	1.6	2.3
Stress-70 protein, mitochondrial precursor	36	2.5	2.1	1.3
	37	1.7	2.3	3.0
T-complex protein 1, epsilon subunit	47	4.4	3.9	5.1
Triosephosphate isomerase	388	2.3	2.2	2.5
	394	2.5	1.6	1.7
Tropomyosin alpha 3 chain	310	5.4	3.6	3.4
	312	4.6	3.7	1.7
Tubulin beta-5 chain	286	0.5	0.3	0.3
	292	0.9	1.0	0.4
Ubiquinol-cytochrome C reductase complex core protein 1	116	1.6	2.5	3.2
Vimentin	80	2.8	0.8	n.d.
	93	1.7	1.0	n.d.
	154	0.9	1.1	4.1
	398	0.7	0.8	2.8

Data are reported as the relative intensity of IEF-SDS-PAGE, CBB-250 stained gel spots after 8, 16 and 24h incubation with 25μM cisplatin, compared with untreated cells. Data for each time point are the average obtained for 3 gels. The Table lists 34 regulated proteins identified by MALDI-TOF which were detected as 62 isoelectric forms. n.d (not detected)

### Systems biology of mass spectrometry data

**Figure 1:**
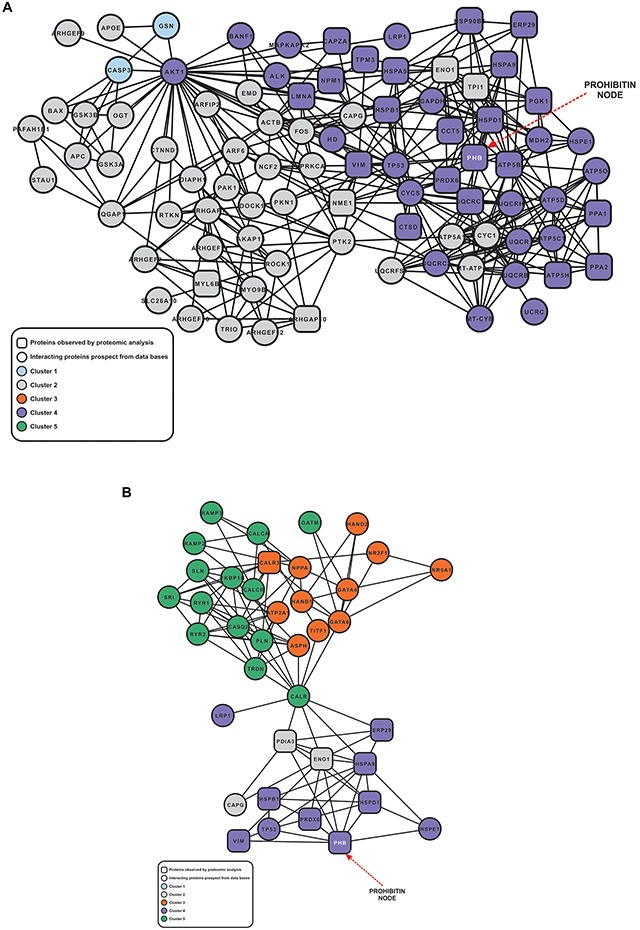
Subnetwork of proteins associated different cellular process The node shapes as well as its colors are described in the Figure's inset. The node representing PHB is indicated in the network by a red dashed arrow. **(A)** Subnetwork of proteins associated with the generation of precursor metabolites and energy, oxidative phosphorylation, regulation of apoptosis, cell motility. **(B)** Subnetwork of proteins associated with protein folding, muscle contraction, cellular di-, trivalent inorganic cation homeostasis, and cell motility.

A systems biology approach to the differentially accumulated proteins showed that cisplatin treatment initiated several different biological processes that led to a cell response resulting in cell survival, as indicated in the network of proteins identified by mass spectrometry and protein interactome prospection (Supplementary Material 1). The application of cluster analysis indicated that the network is composed of five major clusters (Supplementary Material 1) associated with actomyosin assembly/disassembly, cytoskeleton organization, cell cycle regulation and small GTPase-mediated signal transduction, and regulation of transcription and cell differentiation (Supplementary Material 1 and 2). However, only two of the observed subnetworks (Figure 1A and 1B) contain most of the proteins identified by proteomic analysis (Table 1). Additionally, cluster analysis indicated that the proteins belonging to Cluster 4 (Supplementary Material 2) appear to act in association with Clusters 1 and 2 (Figure 1A) and also with Clusters 3 and 5 (Figure 1B), thus suggesting potentially interlinked biologic processes. The biologic processes observed in both subnetworks include different mechanisms associated with the generation of metabolites and energy, oxidative phosphorylation, calcium ion homeostasis, response to unfolded proteins and ER-nuclear signaling pathways, regulation of cell death and apoptosis, among others (Supplementary Material 2). The degree number of nodes belonging to both subnetworks was 12.0 and 8.0, respectively, for PHB in comparison to the average degree of both subnetworks (7.82 and 5.84, respectively). The above-average degree values of PHB suggest that this protein could be a major hub within both subnetworks.

### Cisplatin stimulates prohibitin accumulation as well as cell death in melanoma cells other than LB373 cells

**Figure 2:**
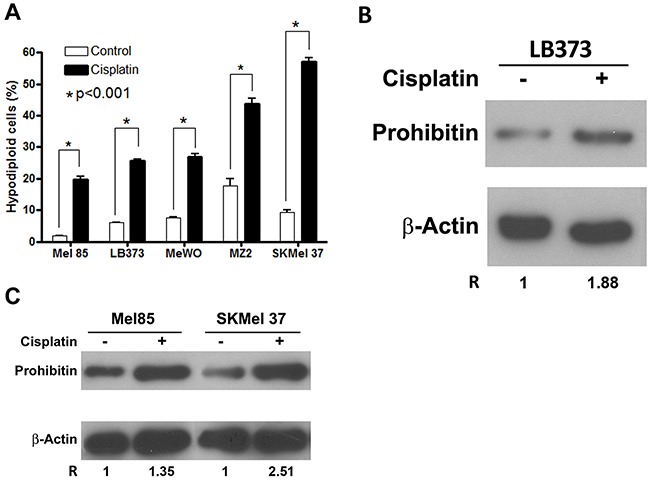
Cisplatin induces prohibitin accumulation and cell death in different melanoma cells Cells were treated with 25 μM of cisplatin for 24 h. **(A)** Cisplatin treatment induced increase in the frequency of hypodiploid cells in Mel 85, LB373, MeWO, MZ2Mel and SKMel 37 cell lines. Cell death was also determined using both JC-1 staining and annexin V/PI double staining for LB373 cell line (Supplementary Figure 2). Protein extracts of LB373 **(B)**, Mel85 and SKMel 37 **(C)** cells, either treated (+) or not (−) with cisplatin were separated in SDS-PAGE and blotted for analysis of PHB and beta-actin accumulation. Protein accumulation was measured by densitometry using ImageJ software. The ratio of PHB/beta-actin accumulation was fixed as 1 for all the control conditions (non-treated cells). Values > 1 indicate relative accumulation of PHB after cisplatin treatment. Representative experiment of three independent assays.

Five metastatic melanoma cell lines were treated with the same protocol as used in the proteomic approach. All melanoma cells showed a different sensitivity to cisplatin (Figure 2A). We chose the most resistant cell line, Mel 85, and the most sensitive one, SKMel 37, together with LB373, to monitor PHB expression after cisplatin treatment. All these cell lines accumulated PHB after cisplatin treatment (Figure 2B and 2C). LB373 had a 1.88-fold accumulation of PHB after cisplatin treatment (Figure 2B), similar as seen in the IEF-SDS-PAGE (Table 1). Mel 85 showed a 1.35 fold in PHB accumulation while SKMel 37 presented a > 2-fold accumulation of this protein (Figure 2C). Interestingly, there is a correlation between PHB accumulation after cisplatin treatment and cell death levels (Figure 2). Also, cisplatin induced loss of mitochondrial membrane potential, as evaluated by the ratio of cytoplasmic and monomeric (green)/ mitochondrial and aggregated (red) fluorescent dye JC-1, leading to the induction of apoptosis of LB373 melanoma cells (Supplementary Figure 2).

### Prohibitin accumulates in response to different stressing stimuli in melanoma cell

**Figure 3:**
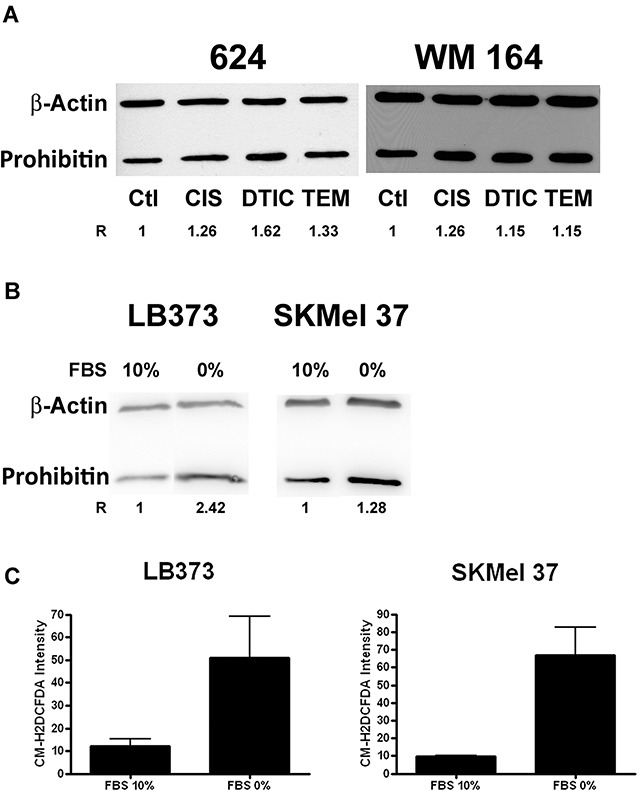
Prohibitin accumulates upon different stressing stimuli **(A)** Cells were treated with 25 μM of cisplatin (CIS) for 24 h, 200 μM of dacarbazin (DTIC) for 48 h or 800 μM of temozolomide (TEM) for 48 h or left untreated. As for Figure 3, protein extracts of melanoma cells under the different control and experimental conditions were analyzed for PHB accumulation, as compared to a protein loading control (beta-actin). R indicates the relative accumulation of PHB. **(B)** Prohibitin also accumulates under serum starvation. LB373 and SKMel 37 cell lines were cultured with (10%) or without (0%) fetal bovine serum (FBS) for 24 h. Prohibitin accumulated in both cell lines upon starvation **(C)**. FBS deprivation induced ROS production, as measured by flow cytometry using the general oxidative stress indicator CM-H2DCFDA in LB373 and SKMel 37 cell lines. Representative experiment of three independent assays.

Dacarbazine and temozolomide, as well as cisplatin, increased PHB protein accumulation in the 624 and WM 164 melanoma cells. The melanoma cell line 624 seemed to be more responsive to these drugs than WM 164 (Figure 3A). Also, fetal bovine serum deprivation for 24 h was sufficient to induce PHB overexpression in LB373 and SKMel 37 melanoma cells (Figure 3B) and PHB overexpression was accompanied by ROS production in these cells (Figure 3C).

### Prohibitin is co-localized with mitochondria after cisplatin treatment

**Figure 4:**
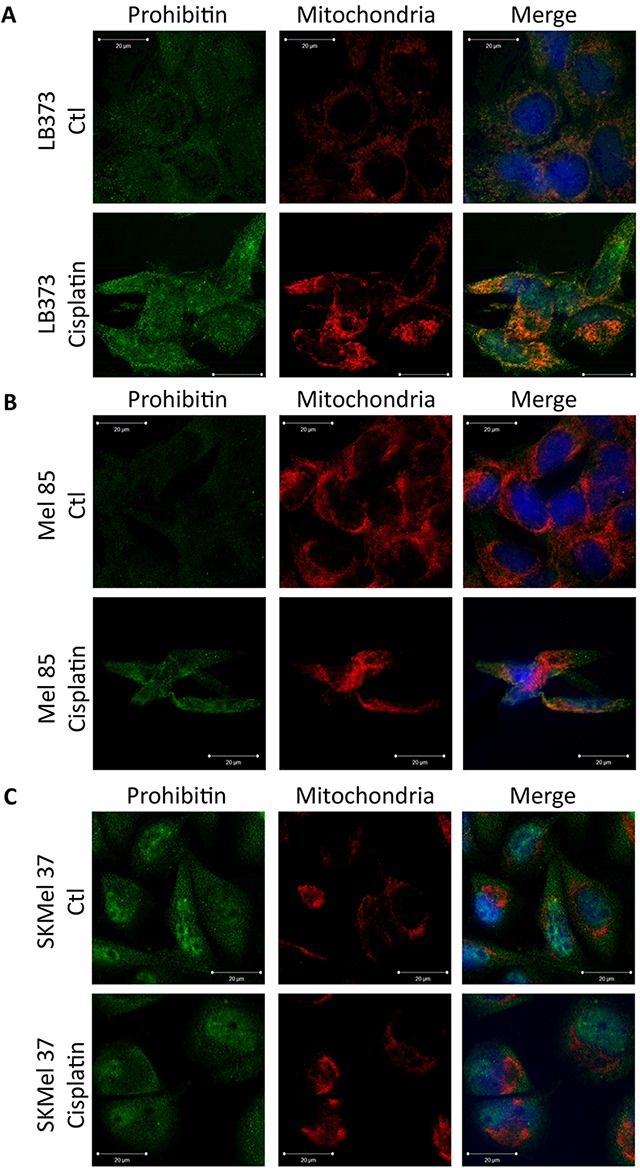
Differential subcellular localization of prohibitin in melanoma cell lines Cells were either treated with 25 μM of cisplatin or left untreated (control) for 24 h and stained as described under Materials and Methods for mitochondria (using Mitotracker, in red), PHB (using Alexa-488 labeled antibodies, green) and nucleus (using Hoechst 33258, blue) and further analyzed using a confocal microscope. **(A)** Prohibitin was found in the nucleus and colocalized with the mitochondria, even without cisplatin treatment in the LB373 melanoma cell line. **(B)** In the Mel 85 melanoma cell line, PHB was also found in the nucleus, and colocalized with the mitochondria after cisplatin treatment. **(C)** In the SKMel 37 melanoma cell line, PHB was found in the nucleus, but could not be seen within the mitochondria. Scale bar in white (5μm). Representative experiment of three independent assays.

Prohibitin can be detected in the nucleus, in the mitochondria and in the plasma membrane. In LB373 (Figure 4A), Mel 85 (Figure 4B) and SKMel 37 cells (Figure 4C), PHB was also detected in nuclear puncta. This pattern was associated with the PHB1 colocalization with E2F family members [13]. Prohibitin was not observed in the nucleolus. In the cytoplasm, PHB was detected mostly in the mitochondria of LB373 and Mel 85 melanoma cells (Figure 4A and 4B), but in SKMel 37 cells there was no colocalization of PHB1 with mitochondria (Figure 4C). All images show evidence of PHB accumulation after cisplatin treatment, but cisplatin treatment did not modify PHB's subcellular distribution (Figure 4A).

### Prohibitin accumulation protects against cisplatin- and tunicamycin-induced cell death

**Figure 5:**
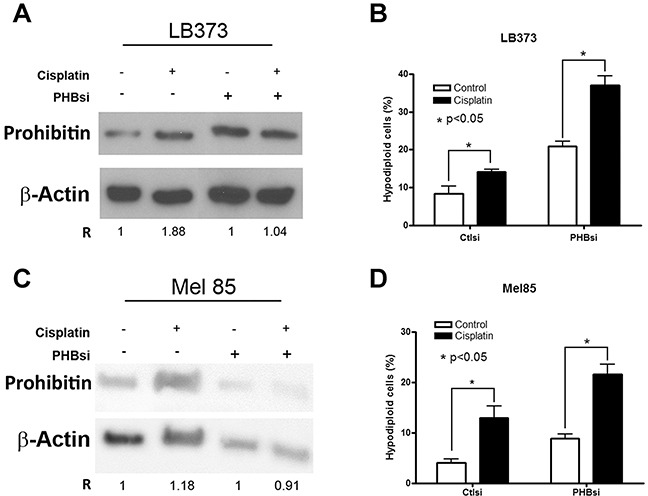
Prohibitin accumulation protects against cisplatin-induced cell death Cells were treated with 25 μM of cisplatin for 24 h. LB373 **(A and B)** and Mel 85 **(C and D)** are more sensitive to cisplatin treatment when PHB is partially knocked down using specific small interference RNA (siRNA). Note that PHB siRNA, but not ctl siRNA, avoided the accumulation of PHB after cisplatin treatment (A and C). As for Figures 3 and 4, the ratio was determined as 1 for each control condition (absence of cisplatin). The relative frequency of hypodiploid cells was used as a measure for cell death. Increased death was observed in both cell lines treated with PHBsi RNA (B and D), which also led to a significant sensitivity of cells to cisplatin treatment. (PHBsi, small interference RNA to PHB; Ctlsi, nonspecific small interference RNA). Representative experiment of three independent assays.

Since many reports describe PHB as a chaperone important for mitochondrial homeostasis [14], we expected that its accumulation would protect cells from cell death. Therefore, we knocked PHB down in order to see if its loss would sensitize cells to stress stimuli such as treatment with cisplatin and a UPR inducer, tunicamycin. The knock-down used only blocked PHB accumulation after cisplatin treatment in the LB373 and Mel 85 melanoma cell lines. In both cell lines, the loss of PHB accumulation (Figure 5A and 5C) was sufficient to sensitize these cells to cisplatin treatment (Figure 5B and 5D). Interestingly, siRNA treatment sensitized these cells even in the absence of cisplatin treatment, suggesting that PHB is indeed important for cell homeostasis.

**Figure 6:**
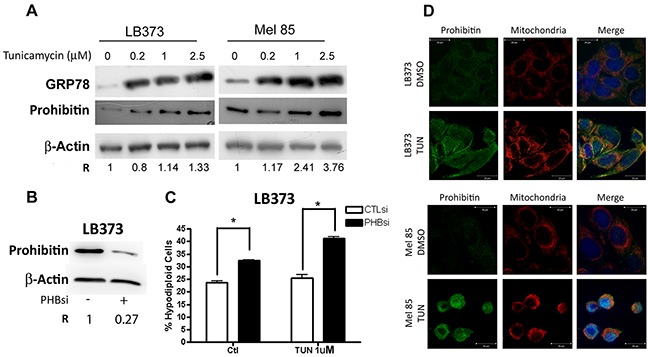
Prohibitin accumulation protects against tunicamycin-induced cell death Cells were either treated 0.2 μM, 1.0 μM or 2.5 μM of tunicamycin, or left untreated (ctl) for 24 h. **(A)** Tunicamycin can induce PHB accumulation in a dose-dependent manner in LB373 and Mel 85 cell lines. Accumulation of the endoplasmic reticulum stress marker grp78 was used as a positive control for the tunicamycin treatment. **(B)** Prohibitin knock-down using PHBsi led to c.a. 70% reduction in the accumulation of PHB, as compared to control siRNA, as analyzed by western blots. **(C)** Upon PHB knock down, LB373 cells were more sensitive to tunicamycin induced cell death (1.0 μM tunicamycin), altogether the results indicate that PHB accumulation is also part of the unfolded protein response (UPR). **(D)** Analysis of PHB compartmentalization upon tunicamycin treatment was performed as in Figure 5. After tunicamycin treatment, PHB (green) was mainly colocalized with mitochondria (red) in LB373 and Mel 85 cell lines, although PHB could also be found in the nucleus to a certain extent. Scale bar in white (5μM). Representative experiment of two independent assays.

Another stressing stimulus is the accumulation of unfolded proteins in the lumen of the endoplasmic reticulum (ER), induced by agents such as tunicamycin. The induction of ER stress is followed by a translation blockade and degradation of misfolded proteins, which are cleared from the ER lumen. If clearance is not possible, a death signal is triggered by the cell. Figure 6 shows that tunicamycin treatment induced ER stress in the LB373 and Mel 85 cell lines, as indicated by the accumulation of the ER stress marker GRP78 (glucose-regulated protein of 78 kDa/immunoglobulin heavy-chain-binding protein) [15]. Tunicamycin induced PHB accumulation in a dose-dependent manner (Figure 6A). Under these conditions, knocking down PHB expression sensitized the LB373 melanoma cell line to tunicamycin treatment (Figure 6B and 6C). Also, since PHB is localized within mitochondria after tunicamycin treatment, the data suggest that the protective role of PHB after tunicamycin treatment is associated with its mitochondrial function (Figure 6D).

## DISCUSSION

New approaches are necessary to treat tumors with high genetic heterogeneity such as melanomas in metastatic disease, so that patients may have an increased life expectancy since conventional therapy usually has a poor outcome. New treatments for melanoma involving the use of targeted agents such as vemurafenib (Braf^V600E^ inhibitor) and ipilimumab (anti-CTLA4) are cost-prohibitive in many low- to middle-income countries. Less expensive alternatives are, therefore, necessary since neither the public health system nor the patient can afford targeted therapies, unless, of course, financial agreements are made with the pharmaceutical industry. Improving conventional chemotherapeutic regimens through innovation or combination approaches seems to be a plausible strategy to enhance access to treatment. Prohibitin may be an interesting target for new therapies. Recently, CRAF/PHB interaction has been shown as an important factor for vemurafenib resistance in melanoma patients [16]. As the cost of vemurafenib treatment is a relevant issue to be considered, it is important to figure out whether PHB accumulation renders cells resistant to potentially cost-effective therapeutic options, such as dacarbazine/temozolomide and cisplatin. New drugs based in the natural compound flavagline has the ability to block CRAF/PHB interaction [17, 18] and could be used as a potential PHB inhibitor as pharmacological treatment.

Cisplatin-induced cell death depends on the production of ROS, which in turn triggers a variety of prosurvival pathways that render part of the cells treatment-resistant. Overcoming these mechanisms that promote survival and the selection of more resistant cells will be useful to induce chemosensitization. In mitochondria, cisplatin induces ROS and nitric oxide (NO) production, activation of mitochondrial outer membrane polarization via BCL-2 family activation, and, in some models, activation of ER stress [19–22]. Melanoma cells that undergo cisplatin treatment usually present an efficient damage protection system [23] since cisplatin is not effective in melanoma patients.

The proteomic approach applied to the cisplatin-treated LB373 melanoma cell line (Supplementary Figure 3) was useful in the identification of processes triggered by cell exposure to cisplatin in a time-dependent manner. Consistent results were obtained regarding the accumulation of some expected electrophoretic forms such as alpha enolase, vimentin and triosephosphate isomerase [24], involved in the cellular response to cisplatin treatment. HSP60, HSP70.1 and the ER stress-related 78 kDa GRP78 are some of the accumulated proteins which are also related to stress responses [25, 26]. In the present study we have focused our attention on PHB since it accumulated consistently with time (Supplementary Figure 4) and is related to the ROS increase in many cancer models [27–31]. The system biology approach showed that different biological processes are modified after cisplatin treatment. Some of them, such as generation of precursor metabolites and energy, oxidative phosphorylation, mitochondrial organization and biogenesis and response to unfolded protein are part of the adaptation to an elevated ROS environment where PHB accumulation is also part of the adaptation, since PHB can work as a molecular chaperone preventing protein misfolding, ROS production and mitochondria biogenesis [32] (Figure 1A and 1B).

LB373 cells were treated with increasing concentrations of cisplatin for 24 h in order to determine the EC50 value of the drug. The kinetics of cell death, exposing the LB373 melanoma cells at the EC50 value of cisplatin, was also determined. Cell death was monitored by loss of propidium iodide exclusion (Supplemental Figure 1) and concentration and time was used to perform the proteomic assay (Supplementary Figure 3 and 4). Cisplatin treatment led to PHB accumulation in LB373 (Figure 2B), Mel 85 and SKMel 37 (Figure 2C) melanoma cells, while inducing cell death (Figure 2A). In Figure 2, different cell lines were exposed to a fixed concentration of cisplatin (25 μM for 24 h) and evaluated the frequency of hypodiploid cells as a proxy for cell death. Under these conditions, around 20% of Mel85 cells, 25% of LB373 cells and 50% of SKMel37 cells were hypodiploid. The higher the fraction of dying cells, the larger was the accumulation of PHB, as observed in Figure 2. Two scenarios arose from this observation; (i) PHB could be part of a death program, so its accumulation is leading to cell death or (ii) PHB could be protecting cells from death, accumulating in resistant cells. Prohibitin inhibition, in Figures 5 and 6 confirmed that the second scenario is the more correct one. Dacarbazine, temozolomide and FBS deprivation also induced PHB accumulation (Figure 3A and 3B), and FBS deprivation led to an increase of ROS (Figure 3C). Temozolomide induced oxidative stress and glutathione (GSH) inhibition and sensitized melanoma cells to temozolomide-induced cell death [33]. Prohibitin accumulation in response to temozolomide treatment can be part of a stress response system that leads to cell survival. Prohibitin can work in order to maintain protein conformation and organize cellular responses against ROS damage in mitochondria since it can act as a chaperone and as an organizer of membrane spaces [34, 35] in the mitochondria. In general, PHB is part of the response against many threats that lead to ROS increase.

Many reports have identified the subcellular localization of PHB as being in the nucleus, in the cytoplasm, in the mitochondria and in the plasma membrane [36–38]. Each compartment in which PHB is located will define different cellular functions for the molecule. In melanomas, PHB can be detected in the cytoplasm, colocalized with the mitochondria and in the nucleus, in the LB373 and Mel 85 cell lines (Figure 4A and 4B). Interestingly, PHB was not detected in the mitochondria in the SKMel 37 cell line (Figure 4C). This may be explained by the fact that mitochondrial PHB colocalization depends on AKT phosphorylation [37] and the SKMel 37 cell line expresses high PTEN levels and does not have phospho-AKT, in contrast to the LB373 and Mel 85 cell lines (Supplementary Figure 5). Since SKMel 37 does not have PHB within its mitochondria, it keeps accumulating PHB after cisplatin treatment (Figure 2B) in contrast to Mel 85 cells and LB373. Prohibitin on these cells can be colocalized in the mitochondria (Figure 4) and these cells have less accumulation of PHB after cisplatin treatment (Figure 2A and 2B). It is important to point out that SKMel 37 was the cell line most sensitive to cisplatin among all cells analyzed (Figure 2A).

The mitochondrial localization of PHB in melanomas suggests that it is important for resistance to chemotherapeutics, as commented above. In order to test this hypothesis, we carried out experiments in which PHB was knocked down. Western Blot analysis showed that even when a partial knock-down of PHB was obtained (Figure 5A and 5C), LB373 and Mel85 melanoma cells were still able to express PHB but lost the ability to accumulate PHB after cisplatin treatment. Inhibition of accumulation, however, was sufficient to sensitize the LB373 and Mel 85 cell lines against cisplatin treatment (Figure 5B and 5D). Surprisingly, PHB inhibition revert the G1 phase inhibition on Mel 85 cells after cisplatin treatment (Supplementary Figure 6B) while on LB373 cell line, PHB inhibition does not affect cell cycle on a consistent manner (Supplementary Figure 6A and 6C). Taken together, these results suggest that there may be at least two pools of PHB in a given cell, i.e., (i) a basal pool and (ii) a regulatable pool. The latter was sensitive to inhibition of translation with siRNA, and this pool seems to be associated with chemoresistance. Since cells respond to many stimuli that lead to ROS production by inducing PHB expression, PHB may be involved in the response against ROS in order to protect the mitochondria.

Another stress pathway examined here was the unfolded protein response. One of the UPR markers is the ER stress-related 78 kDa GRP78 which also accumulated after cisplatin treatment in the proteome of the LB373 cell line (Supplementary Figure 3 and Table). Tunicamycin induced PHB accumulation, indicating that PHB is part of the UPR in both LB373 and Mel 85 cell lines (Figure 6A). Prohibitin accumulation protected the LB373 cell line against tunicamycin-induced cell death since its knock- down sensitized this cell line to tunicamycin (Figure 6C). Differently from cisplatin, tunicamycin was not able to change cell cycle profile on LB373 cells after PHB inhibition (Supplementary Figure 6A).

Prohibitin cannot be detected in the ER [39] but can be found in the mitochondria after tunicamycin treatment, suggesting that its accumulation protects mitochondria during UPR. It is not clear, however, how the unfolded protein response stimulates PHB accumulation. Indeed, UPR is characterized by a decrease in translational activity, although selected proteins do accumulate upon the activation of UPR [40]. One of the mechanisms involved in UPR is Gadd153 activation, which can induce ROS production and lead to cell death [41]. In this case, PHB could protect mitochondria from ROS damage. Another possibility is that UPR can induce G1 arrest by inhibiting cyclin D1 translation [42], making retinoblastoma protein phosphorylation and E2F1 release more difficult. Since PHB is also an E2F1 repressor, its accumulation by tunicamycin treatment would contribute to E2F1 repression and blockade of the cell cycle in G1.

Recently, a new subpopulation of melanomas with high mitochondrial activity and ROS production, characterized by histone demethylase JARID1b overexpression, has been reported to be extremely resistant to chemotherapy and to be responsible for tumor repopulation [43]. These JARID1b-high cells in melanomas do not show differences in the expression of genes related to cell survival such as Bcl-2, but they accumulate proteins related to mitochondrial activity [43, 44]. Prohibitin can be part of the adaptation of the JARID1b-high cells against ROS by protecting the mitochondria, thus representing a potential target against subpopulations of melanoma cells that lead to treatment failure.

## CONCLUSION

The present results indicate that PHB is necessary for cell adaptation against cellular stress. In general, PHB expression is critical for cell survival in an imbalanced redox environment. Understanding the mechanisms that control PHB transcription and translation will be critical to target this molecule.

## MATERIALS AND METHODS

### Cell lines

The human melanoma metastatic cell lines SKMel 37 [45], Skmel 28 [46], 624 [47], WM 164 [48], MeWO [49], LB373 [50], Mel 85 [51] and MZ2Mel [51] were used in this study. SKMel 28, LB373, MeWO and Mel 85 were cultured in RPMI 1640, SKMel 37 was cultured with Minimum Essential Medium Eagle (MEM) and MZ2Mel 624 and WM 164 were cultured in Dulbecco's Modified Eagle's Medium (DMEM). All culture media were supplemented with 10% fetal bovine serum (FBS). Skmel 37, SKmel 28, MeWO, LB373, Mel85 and MZ2 melanoma cells were kindly provided by Ludwig Cancer Research Institute. 624 and WM 164 melanoma cells were kindly provided by Moffitt Cancer Research Center.

### Cell treatment

For each treatment, 3×10^4^ cells were plated onto a 12 well plate 24 h prior to the treatment and incubated in a 37°C and 5% CO_2_ incubator, except for the proteomic assay. The concentration and time used for each treatment carried out at 25°C were: cisplatin (25 μM for 24 h), dacarbazine (200 μM for 48 h), temozolomide (800 μM for 48 h) and tunicamycin (0.2, 1.0 and 2.5 μM for 24 h).

For the proteomic assay, 1×10^7^ cells were plated onto a 175 cm^2^ plate 24 h prior to treatment with cisplatin. Cells were treated with 25 μM cisplatin for 8 h, 16 h and 24 h.

### Isoelectric focusing (IEF) and SDS-PAGE

An IPG-Phor electrophoretic system was used with 18 cm pH 4-7 gradient strips (GE Healthcare). The strip was reduced with 65 mM DTT and alkylated with 100 mM iodoacetamide. One mg protein was applied during rehydration (Rabilloud, 1994). Electrophoresis was carried out at 50 mA/strip and 150 V for 1 h, 500 V for 1 h, 1000 V for 1 h, and 8000 V until 70,000 Vh were accumulated. SDS-PAGE was carried out in an EttanDALTsix electrophoretic system (GE Healthcare Life Sciences) using homogeneous 12.5% polyacrylamide gels of 1.5 mm thickness. The IEF strip was placed on top of the gel with 0.5% agarose. Electrophoresis was carried out at 20 mA/gel, 300 V, 6 W, at 10°C until the bromophenol left the gel after about 15 h [11]. Gels were fixed in 40% v/v ethanol containing 10% v/v acetic acid for 1 h, washed in water and stained with 0.1% w/v colloidal CBB-250 (Serva, Germany) containing 2% w/v phosphoric acid, 10% w/v ammonium sulfate, and 20% v/v methanol for 24 h. After staining, gels were briefly washed in 25% v/v methanol and stored in 20% w/v ammonium sulfate at 4°C until use.

Gels were scanned with the ImageScanner acquisition system (GE Health Care) using ImageMaster-2D software. Comparisons between 2 D maps of cells treated or not with cisplatin were made between spots of three replicate gels of cells treated or not with cisplatin. Reproducibility of the location of the same spots on pairs of gels was 86 and 91% and the number of spots not detected in other gels was 14, 61, and 31. These spots were of low intensity. Variations in pI and molecular mass were 0.3 and 2.0%, respectively. Ninety percent or more of all three spots detected at the same location had high staining intensity of >400. The precision of the measured intensity was <±40% for 20 spots in triplicate gels. Therefore plus or minus 2-fold was used to define protein accumulation or reduction and used to compare the same spot before and after cisplatin treatment.

Spots were cut out of the gel and eluted with 0.1 M NH_4_CO_3_, pH 8.0, containing 50% acetonitrile (CAN) until the stain was extracted. The extract was rehydrated with 5 μl of 1 M NH_4_CO_3_containing 0.4μg trypsin (Promega) and incubated for 24 h at 37°C. Peptides were eluted 3 times with 100 μl of 50% ACN containing 2.5% trifluoroacetic acid (TFA). The peptides were desalted on C_18_ resin in a pipette tip and analyzed with a MALDI-TOF spectrometer (model LR, Micromass). Peptides were incorporated into a matrix with 1% CHCA, 50% ACN and 0.2% TFA, evaporated and ionized. The mass spectrometer was calibrated with the following peptides: angiotensin I (1297.51 Da), ACTH 1-17 (2094.35 Da), ACTH 18-39 (2466.74 Da), ACTH 7-38 (3660.22 Da), and bovine insulin (5734.60 Da). Experimental details are given in Souza *et al* [52].

### Databank searches

Ions identified by MS were analyzed with the MS-Fit tool (Protein Prospector – http://prospector.ucsf.edu) using the Swiss-Prot databank for human-mouse proteins. The parameters used for the search were 0.2 Da for permitted mass error and one missed cleavage site for trypsin hydrolysis specificity. Proteins were identified on the basis of minimum sequence coverage of more than 15%. Functional protein classification was based on level 5 of the Gene Ontology classification, available at http://source-search.princeton.edu.

### Systems biology analysis

The data obtained from mass spectrometry analysis were used as input in the metasearch engines STRING 9.1 [53]. The following prospection parameters were used in the STRING: all prediction methods enabled, excluding text mining and degree of confidence 0.400. The protein-protein interaction network was analyzed in terms of cluster structure and node centralities with Cytoscape 2.8.3 [54, 55].

The major cluster composition of the protein-protein interaction network was created with Molecular Complex Detection (MCODE) plugin [56] based on the following parameters: degree cutoff, 2; node score cutoff, 0.2; k-core, 2; and maximum network depth, 100; fluff option enabled with node density cutoff, 0.1; and haircut option enabled. As a result, each cluster generates a degree of connection in a given group of nodes, also called value of “cliquishness” (Ci). In this respect, score values where Ci > 3.0 were considered to be the cutoff.

The major biological processes associated with each cluster were accessed using the plugin Biological Network Gene Ontology (BiNGO) 2.44 [57]. The degree of functional enrichment for a given gene ontology category was quantitatively assessed (p-value) using a hypergeometric distribution [58]. Multiple test correction was also assessed by applying the false discovery rate (FDR) algorithm, which was fully implemented in BiNGO software at an adjusted level of significance of p < 0.05. Degree analysis of nodes was performed with the plugin CentiScape 1.2 [59]. In this analysis, the CentiScaPe algorithm evaluates each network node according to the degree number. Nodes with a high node degree are called hubs and have key regulatory functions in the cell [59].

### Prohibitin knock-down by siRNA

For each inhibition, 6×10^4^ cells were plated onto a 60 mm dish. In Figure 5, 150 nM of PHB siRNA was transfected with 8 μL of lipofectamin 2000® (ThermoFisher). Prohibitin siRNA was incubated with Opti-MEM®, isolated from Lipofectamin for 5 min. Next, PHB siRNA and lipofectamin were incubated together for 20 min for lipofectamin-siRNA complex formation. Cells were then transfected for 6 h, when the Opti-MEM with the lipofectamin-siRNA complex was removed from the dish and the respective cell culture medium was added. After 48 h, cells were plated for further experiments.

In Figure 6, the same siRNA protocol was used except that oligofectamin® (ThermoFisher) was used instead of lipofectamin 2000®.

### Flow cytometry assay

Cells were plated according to each experiment and then were detached from the plate, washed with PBS and resuspended in 70% ethanol for 2 h at room temperature for fixation. Cells were then washed once with PBS and incubated in 200 μL of propidium iodide solution (0.1% Triton X-100, 200 μg/ml of RNAse A and 20 μg/ml of propidium iodide) for 30 min at room temperature, protected from light. About 1×10^4^ cells were analyzed with a FACScalibur flow cytometer (Becton Dickinson®). The Sub-G1 content was used to estimate cells that were in the cell death process.

### Protein extraction and western blot

For each experiment, 6×10^4^ cells were plated per well on a 6 well plate and then treated according to each condition. Cells were then trypsinized and centrifuged at 370 *g* for 2 min. The cell pellet was dispersed in NP40 lysis buffer with protease inhibitors (1 mM DTT, 0.1 mM PMSF and 5 μg/ml aprotinin). After the cells were homogenized, they were left to stand at 4°C for 30 min and the homogenate was centrifuged at 4°C for 15 min at 15600 *g*. The supernatant obtained by centrifugation of this extract was aliquoted and stored at -20°C. The protein content of the extract was measured with the BCA reagent kit (ThermoFisher®).

About 50 μg of proteins were separated on polyacrylamide gel containing 0.375 M Tris, pH 8.8, 0.1% SDS, 10% acrylamide, 0.03% ammonium persulfate (APS), and 0.06% N,N,N′,N′-tetramethylethyilenediamine (TEMED). The stacking gel composition was 0.125 M TRIS, 0.1% SDS, 4% acrylamide, 0.045% APS and 0.06% TEMED, pH 6.8. The separated protein extract was then transferred to a hydrophobic polyvinylidene difluoride (PVDF) membrane in 25 mM Tris, 20% methanol (v/v) for one hour at 100 V and 4°C. The nonspecific sites of the membrane were blocked with 5% fat-free milk in 0.1% PBS-Tween for 1 h at room temperature and the membrane was incubated with the primary antibody overnight at 4°C. The membrane was then washed three times for 10 min and subsequently incubated with the peroxidase-conjugated secondary antibody for one h at room temperature. Spot intensity measurement was performed using ImageJ® software.

### Confocal microscopy

1×10^4^ Cells were plated onto a 24 well plate over a 30 mm coverslip. Mitotracker Red CMXRos (Life Technologies®) was used to label the mitochondria. Mitotracker Red was diluted in each cell culture medium for 20 min at a concentration of 500 nM and incubated at 37°C in the presence of 5% CO_2_. Cells were washed with PBS and then fixed in 4% paraformaldehyde in PBS for 15 min. After three washes with PBS, 0.2% Triton X-100 in PBS was added for 5 min for cell permeabilization. Nonspecific sites were blocked with PBS with 5% BSA for 1 h. Mouse monoclonal antibody to PHB MS-261-P0 (Thermofisher®) (4 μg/mL), was incubated overnight at 4°C in PBS with 5% BSA. Secondary antibody conjugated with anti-mouse Alexa Fluor 488 (Thermofisher®) (4 μg/mL) and the nuclear marker Hoechst 33258® was incubated for 1 h at room temperature in PBS containing 5% BSA. The analysis was performed using a Zeiss LSM 510® Meta/ UV confocal microscope.

### Cell starvation and reactive oxygen species assay

3×10^4^ cells per well were plated onto a 12 well plate. After 24 h, cells were washed twice with PBS and then cultured with normal FBS concentration (10%) or without FBS in their respective culture media for 24 h. Cells were then washed twice with Hank's Balanced Salt Solution (HBSS) and incubated with 5 μM CM-H2DCFDA (Life Technologies®) in HBSS for 30 min at 37°C in a 5% CO_2_ incubator. After incubation, cells were washed with PBS and returned to the 37°C and 5% CO_2_ incubator in their respective culture media for 30 min. About 1×10^4^ cells were analyzed with a FACScalibur flow cytometer (Becton Dickinson®) to measure ROS levels in each cell line.

## SUPPLEMENTARY FIGURES



## Abbreviations

MTIC: 5-[3-methyl-triazen-1-yl]-imidazole-4-carboxamide
CDDP: Cisplatin
DTIC: Dacarbazine
DDB2: DNA-binding protein 2
GSH: Glutathione
MAPK: Mitogen-Activated Protein Kinase
NO: Nitric Oxide
NER: Nucleotide Excision Repair
PHB: Prohibitin
PHB2: Prohibitin 2
ROS: Reactive Oxygen Species
UPR: Unfolded Protein Response
XPC: Xeroderma Pigmentosum Complementation group C
TMZ: Temozolomide

## References

[1] Chapman PB, Hauschild A, Robert C, Haanen JB, Ascierto P, Larkin J, Dummer R, Garbe C, Testori A, Maio M, Hogg D, Lorigan P, Lebbe C (2011). Improved survival with vemurafenib in melanoma with BRAF V600E mutation. N Engl J Med.

[2] Hodi FS, O’Day SJ, McDermott DF, Weber RW, Sosman JA, Haanen JB, Gonzalez R, Robert C, Schadendorf D, Hassel JC, Akerley W, van den Eertwegh AJ, Lutzky J (2010). Improved survival with ipilimumab in patients with metastatic melanoma. N Engl J Med.

[3] Larkin J, Chiarion-Sileni V, Gonzalez R, Grob JJ, Cowey CL, Lao CD, Schadendorf D, Dummer R, Smylie M, Rutkowski P, Ferrucci PF, Hill A, Wagstaff J (2015). Combined Nivolumab and Ipilimumab or Monotherapy in Untreated Melanoma. N Engl J Med.

[4] Khayat D, Bernard-Marty C, Meric JB, Rixe O (2002). Biochemotherapy for advanced melanoma: maybe it is real. J Clin Oncol.

[5] Eton O, Legha SS, Bedikian AY, Lee JJ, Buzaid AC, Hodges C, Ring SE, Papadopoulos NE, Plager C, East MJ, Zhan F, Benjamin RS (2002). Sequential biochemotherapy versus chemotherapy for metastatic melanoma: results from a phase III randomized trial. J Clin Oncol.

[6] Cullen KJ, Yang Z, Schumaker L, Guo Z (2007). Mitochondria as a critical target of the chemotheraputic agent cisplatin in head and neck cancer. J Bioenerg Biomembr.

[7] Barckhausen C, Roos WP, Naumann SC, Kaina B (2014). Malignant melanoma cells acquire resistance to DNA interstrand cross-linking chemotherapeutics by p53-triggered upregulation of DDB2/XPC-mediated DNA repair. Oncogene.

[8] Federici C, Petrucci F, Caimi S, Cesolini A, Logozzi M, Borghi M, D’Ilio S, Lugini L, Violante N, Azzarito T, Majorani C, Brambilla D, Fais S (2014). Exosome release and low pH belong to a framework of resistance of human melanoma cells to cisplatin. PLoS One.

[9] Li W, Melton DW (2012). Cisplatin regulates the MAPK kinase pathway to induce increased expression of DNA repair gene ERCC1 and increase melanoma chemoresistance. Oncogene.

[10] Marullo R, Werner E, Degtyareva N, Moore B, Altavilla G, Ramalingam SS, Doetsch PW (2013). Cisplatin induces a mitochondrial-ROS response that contributes to cytotoxicity depending on mitochondrial redox status and bioenergetic functions. PLoS One.

[11] Martins NM, Santos NA, Curti C, Bianchi ML, Santos AC (2008). Cisplatin induces mitochondrial oxidative stress with resultant energetic metabolism impairment, membrane rigidification and apoptosis in rat liver. J Appl Toxicol.

[12] Calvani M, Comito G, Giannoni E, Chiarugi P (2012). Time-dependent stabilization of hypoxia inducible factor-1α by different intracellular sources of reactive oxygen species. PLoS One.

[13] Rastogi S, Joshi B, Dasgupta P, Morris M, Wright K, Chellappan S (2006). Prohibitin facilitates cellular senescence by recruiting specific corepressors to inhibit E2F target genes. Mol Cell Biol.

[14] Merkwirth C, Martinelli P, Korwitz A, Morbin M, Brönneke HS, Jordan SD, Rugarli EI, Langer T (2012). Loss of prohibitin membrane scaffolds impairs mitochondrial architecture and leads to tau hyperphosphorylation and neurodegeneration. PLoS Genet.

[15] Andruska N, Zheng X, Yang X, Helferich WG, Shapiro DJ (2015). Anticipatory estrogen activation of the unfolded protein response is linked to cell proliferation and poor survival in estrogen receptor α-positive breast cancer. Oncogene.

[16] Doudican NA, Orlow SJ (2017). Inhibition of the CRAF/prohibitin interaction reverses CRAF-dependent resistance to vemurafenib. Oncogene.

[17] Polier G, Neumann J, Thuaud F, Ribeiro N, Gelhaus C, Schmidt H, Giaisi M, Kohler R, Muller WW, Proksch P, Leippe M, Janssen O, Desaubry L (2012). The natural anticancer compounds rocaglamides inhibit the Raf-MEK-ERK pathway by targeting prohibitin 1 and 2. Chem Biol.

[18] Basmadjian C, Thuaud F, Ribeiro N, Desaubry L (2013). Flavaglines: potent anticancer drugs that target prohibitins and the helicase eIF4A. Future Med Chem.

[19] Kroemer G, Galluzzi L, Brenner C (2007). Mitochondrial membrane permeabilization in cell death. Physiol Rev.

[20] Brenner C, Grimm S (2006). The permeability transition pore complex in cancer cell death. Oncogene.

[21] Godoy LC, Anderson CT, Chowdhury R, Trudel LJ, Wogan GN (2012). Endogenously produced nitric oxide mitigates sensitivity of melanoma cells to cisplatin. Proc Natl Acad Sci U S A.

[22] Yu F, Megyesi J, Price PM (2008). Cytoplasmic initiation of cisplatin cytotoxicity. Am J Physiol Renal Physiol.

[23] Biroccio A, Benassi B, Amodei S, Gabellini C, Del Bufalo D, Zupi G (2001). c-Myc down-regulation increases susceptibility to cisplatin through reactive oxygen species-mediated apoptosis in M14 human melanoma cells. Mol Pharmacol.

[24] Petrak J, Ivanek R, Toman O, Cmejla R, Cmejlova J, Vyoral D, Zivny J, Vulpe CD (2008). Déjà vu in proteomics. A hit parade of repeatedly identified differentially expressed proteins. Proteomics.

[25] Shin BK, Wang H, Yim AM, Le Naour F, Brichory F, Jang JH, Zhao R, Puravs E, Tra J, Michael CW, Misek DE, Hanash SM (2003). Global profiling of the cell surface proteome of cancer cells uncovers an abundance of proteins with chaperone function. J Biol Chem.

[26] Steel R, Cross RS, Ellis SL, Anderson RL (2012). Hsp70 architecture: the formation of novel polymeric structures of Hsp70.1 and Hsc70 after proteotoxic stress. PLoS One.

[27] Zhou P, Qian L, D’Aurelio M, Cho S, Wang G, Manfredi G, Pickel V, Iadecola C (2012). Prohibitin reduces mitochondrial free radical production and protects brain cells from different injury modalities. J Neurosci.

[28] Fujinaga H, Tsutsumi T, Yotsuyanagi H, Moriya K, Koike K (2011). Hepatocarcinogenesis in hepatitis C: HCV shrewdly exacerbates oxidative stress by modulating both production and scavenging of reactive oxygen species. Oncology.

[29] Mörbt N, Tomm J, Feltens R, Mögel I, Kalkhof S, Murugesan K, Wirth H, Vogt C, Binder H, Lehmann I, von Bergen M (2011). Chlorinated benzenes cause concomitantly oxidative stress and induction of apoptotic markers in lung epithelial cells (A549) at nonacute toxic concentrations. J Proteome Res.

[30] Yoo DR, Jang YH, Jeon YK, Kim JY, Jeon W, Choi YJ, Nam MJ (2009). Proteomic identification of anti-cancer proteins in luteolin-treated human hepatoma Huh-7 cells. Cancer Lett.

[31] Da Silva-Azevedo L, Jähne S, Hoffmann C, Stalder D, Heller M, Pries AR, Zakrzewicz A, Baum O (2009). Up-regulation of the peroxiredoxin-6 related metabolism of reactive oxygen species in skeletal muscle of mice lacking neuronal nitric oxide synthase. J Physiol.

[32] Ande SR, Nguyen KH, Padilla-Meier GP, Wahida W, Nyomba BL, Mishra S (2014). Prohibitin overexpression in adipocytes induces mitochondrial biogenesis, leads to obesity development, and affects glucose homeostasis in a sex-specific manner. Diabetes.

[33] Rocha CR, Kajitani GS, Quinet A, Fortunato RS, Menck CF (2016). NRF2 and glutathione are key resistance mediators to temozolomide in glioma and melanoma cells. Oncotarget.

[34] Bourges I, Ramus C, Mousson de Camaret B, Beugnot R, Remacle C, Cardol P, Hofhaus G, Issartel JP (2004). Structural organization of mitochondrial human complex I: role of the ND4 and ND5 mitochondria-encoded subunits and interaction with prohibitin. Biochem J.

[35] Nijtmans LG, de Jong L, Artal Sanz M, Coates PJ, Berden JA, Back JW, Muijsers AO, van der Spek H, Grivell LA (2000). Prohibitins act as a membrane-bound chaperone for the stabilization of mitochondrial proteins. EMBO J.

[36] Kolonin MG, Saha PK, Chan L, Pasqualini R, Arap W (2004). Reversal of obesity by targeted ablation of adipose tissue. Nat Med.

[37] Jiang L, Dong P, Zhang Z, Li C, Li Y, Liao Y, Li X, Wu Z, Guo S, Mai S, Xie D, Liu Z, Zhou F (2015). Akt phosphorylates Prohibitin 1 to mediate its mitochondrial localization and promote proliferation of bladder cancer cells. Cell Death Dis.

[38] Wang S, Fusaro G, Padmanabhan J, Chellappan SP (2002). Prohibitin co-localizes with Rb in the nucleus and recruits N-CoR and HDAC1 for transcriptional repression. Oncogene.

[39] Browman DT, Resek ME, Zajchowski LD, Robbins SM (2006). Erlin-1 and erlin-2 are novel members of the prohibitin family of proteins that define lipid-raft-like domains of the ER. J Cell Sci.

[40] Hiramatsu N, Chiang WC, Kurt TD, Sigurdson CJ, Lin JH (2015). Multiple Mechanisms of Unfolded Protein Response-Induced Cell Death. Am J Pathol.

[41] McCullough KD, Martindale JL, Klotz LO, Aw TY, Holbrook NJ (2001). Gadd153 sensitizes cells to endoplasmic reticulum stress by down-regulating Bcl2 and perturbing the cellular redox state. Mol Cell Biol.

[42] Brewer JW, Hendershot LM, Sherr CJ, Diehl JA (1999). Mammalian unfolded protein response inhibits cyclin D1 translation and cell-cycle progression. Proc Natl Acad Sci U S A.

[43] Roesch A, Vultur A, Bogeski I, Wang H, Zimmermann KM, Speicher D, Körbel C, Laschke MW, Gimotty PA, Philipp SE, Krause E, Pätzold S, Villanueva J (2013). Overcoming intrinsic multidrug resistance in melanoma by blocking the mitochondrial respiratory chain of slow-cycling JARID1B(high) cells. Cancer Cell.

[44] Saito ReF, Tortelli TC, Jacomassi MD, Otake AH, Chammas R (2015). Emerging targets for combination therapy in melanomas. FEBS Lett.

[45] Lloyd KO, Ng J, Dippold WG (1981). Analysis of the biosynthesis of HLA-DR glycoproteins in human malignant melanoma cell lines. J Immunol.

[46] Tarli L, Balza E, Viti F, Borsi L, Castellani P, Berndorff D, Dinkelborg L, Neri D, Zardi L (1999). A high-affinity human antibody that targets tumoral blood vessels. Blood.

[47] Mohapatra S, Coppola D, Riker AI, Pledger WJ (2007). Roscovitine inhibits differentiation and invasion in a three-dimensional skin reconstruction model of metastatic melanoma. Mol Cancer Res.

[48] Gallagher PG, Bao Y, Prorock A, Zigrino P, Nischt R, Politi V, Mauch C, Dragulev B, Fox JW (2005). Gene expression profiling reveals cross-talk between melanoma and fibroblasts: implications for host-tumor interactions in metastasis. Cancer Res.

[49] Du J, Wu J, Fu X, Tse AK, Li T, Su T, Yu ZL (2016). Icairiside II overcomes TRAIL resistance of melanoma cells through ROS-mediated downregulation of STAT3/cFLIP signaling. Oncotarget.

[50] Zhu B, Chen Z, Cheng X, Lin Z, Guo J, Jia Z, Zou L, Wang Z, Hu Y, Wang D, Wu Y (2003). Identification of HLA-A*0201-restricted cytotoxic T lymphocyte epitope from TRAG-3 antigen. Clin Cancer Res.

[51] Traversari C, van der Bruggen P, Van den Eynde B, Hainaut P, Lemoine C, Ohta N, Old L, Boon T (1992). Transfection and expression of a gene coding for a human melanoma antigen recognized by autologous cytolytic T lymphocytes. Immunogenetics.

[52] de Souza GA, Godoy LM, Teixeira VR, Otake AH, Sabino A, Rosa JC, Dinarte AR, Pinheiro DG, Silva WA, Eberlin MN, Chammas R, Greene LJ (2006). Proteomic and SAGE profiling of murine melanoma progression indicates the reduction of proteins responsible for ROS degradation. Proteomics.

[53] Franceschini A, Szklarczyk D, Frankild S, Kuhn M, Simonovic M, Roth A, Lin J, Minguez P, Bork P, von Mering C, Jensen LJ (2013). STRING v9.1: protein-protein interaction networks, with increased coverage and integration. Nucleic Acids Res.

[54] Smoot ME, Ono K, Ruscheinski J, Wang PL, Ideker T (2011). Cytoscape 2.8: new features for data integration and network visualization. Bioinformatics.

[55] Cline MS, Smoot M, Cerami E, Kuchinsky A, Landys N, Workman C, Christmas R, Avila-Campilo I, Creech M, Gross B, Hanspers K, Isserlin R, Kelley R (2007). Integration of biological networks and gene expression data using Cytoscape. Nat Protoc.

[56] Bader GD, Hogue CW (2003). An automated method for finding molecular complexes in large protein interaction networks. BMC Bioinformatics.

[57] Maere S, Heymans K, Kuiper M (2005). BiNGO: a Cytoscape plugin to assess overrepresentation of gene ontology categories in biological networks. Bioinformatics.

[58] Rivals I, Personnaz L, Taing L, Potier MC (2007). Enrichment or depletion of a GO category within a class of genes: which test?. Bioinformatics.

[59] Scardoni G, Petterlini M, Laudanna C (2009). Analyzing biological network parameters with CentiScaPe. Bioinformatics.

