# Supplementary material for: Accumulation of prohibitin is a common cellular response to different stressing stimuli and protects melanoma cells from ER stress and chemotherapy-induced cell death
Source: Oncotarget. 2017 May 11;8(26):43114–29. doi: 10.18632/oncotarget.17810 (PMC5522132; doi:10.18632/oncotarget.17810)
Supplement: Supplementary file 1 [file oncotarget-08-43114-s001.pdf]

Accumulation of prohibitin is a common cellular response to different stressing stimuli and protects melanoma cells from ER stress and chemotherapy-induced cell death

SUPPLEMENTARY MATERIALS AND METHODS

Isoelectric focusing (IEF) and SDS-PAGE

An IPG-Phor electrophoretic system was used with 18 cm pH 4-7 gradient strips (GE Healthcare). The strip was reduced with 65 mM DTT and alkylated with 100 mM iodoacetamide. One mg protein was applied during rehydration (Rabilloud, 1994). Electrophoresis was carried out at 50 mA/strip and 150 V for 1 h, 500 V for 1 h, 1000 V for 1 h, and 8000 V until 70,000 Vh were accumulated. SDS-PAGE was carried out in an EttanDALTsix electrophoretic system (GE Healthcare Life Sciences) using homogeneous 12.5% polyacrylamide gels of 1.5 mm thickness. The IEF strip was placed on top of the gel with 0.5% agarose. Electrophoresis was carried out at 20 mA/gel, 300 V, 6 W, at 10ºC until the bromophenol left the gel after about 15 h [11]. Gels were fixed in 40% v/v ethanol containing 10% v/v acetic acid for 1 h, washed in water and stained with 0.1% w/v colloidal CBB-250 (Serva, Germany) containing 2% w/v phosphoric acid, 10% w/v ammonium sulfate, and 20% v/v methanol for 24 h. After staining, gels were briefly washed in 25% v/v methanol and stored in 20% w/v ammonium sulfate at 4ºC until use.

Gels were scanned with the ImageScanner acquisition system (GE Health Care) using ImageMaster-2D software. Comparisons between 2 D maps of cells treated or not with cisplatin were made between spots of three replicate gels of cells treated or not with cisplatin. Reproducibility of the location of the same spots on pairs of gels was 86 and 91% and the number of spots not detected in other gels was 14, 61, and 31. These spots were of low intensity. Variations in pI and molecular mass were 0.3 and 2.0%, respectively. Ninety percent or more of all three spots detected at the same location had high staining intensity of >400. The precision of the measured intensity was <±40% for 20 spots in triplicate gels. Therefore plus or minus 2-fold was used to define protein accumulation or reduction and used to compare the same spot before and after cisplatin treatment.

Spots were cut out of the gel and eluted with 0.1 M NH_4CO_3, pH 8.0, containing 50% acetonitrile (CAN) until the stain was extracted. The extract was rehydrated with 5 µl of 1 M NH_4CO_3containing 0.4µg trypsin (Promega) and incubated for 24 h at 37ºC. Peptides were eluted 3 times with 100 µl of 50% ACN containing 2.5% trifluoroacetic acid (TFA). The peptides were desalted on C_{18} resin in a pipette tip and analyzed with a MALDI-TOF spectrometer (model LR, Micromass). Peptides were incorporated into a matrix with 1% CHCA, 50% ACN and 0.2% TFA, evaporated and ionized. The mass spectrometer was calibrated with the following peptides: angiotensin I (1297.51 Da), ACTH 1-17 (2094.35 Da), ACTH 18-39 (2466.74 Da), ACTH 7-38 (3660.22 Da), and bovine insulin (5734.60 Da).

Databank searches

Ions identified by MS were analyzed with the MS-Fit tool (Protein Prospector – http://prospector.ucsf.edu) using the Swiss-Prot databank for human-mouse proteins. The parameters used for the search were 0.2 Da for permitted mass error and one missed cleavage site for trypsin hydrolysis specificity. Proteins were identified on the basis of minimum sequence coverage of more than 15%. Functional protein classification was based on level 5 of the Gene Ontology classification, available at http//source.stanford.edu.

JC-1 and Annexin-V

For annexin-V analyses, cells were incubated with Annexin-V/FITC (Invitrogen) in presence of binding buffer (10 mmol/L HEPES, 140 mmol/L NaCl, 2.5 mmol/L CaCl2 x 2H2O, and 0.1% bovine serum albumin). Cell suspension was then transferred to microtubes and incubated on ice, in the dark, for 15 min. 4 µg/mL of propidium iodide were added and samples were immediately analyzed by flow cytometry using FACSCalibur (Beckton and Dickson®, BD Biosciences, San Jose, CA, USA). For JC-1 staining, cells were incubated with 200nM of JC1 in 10% FBS in medium for 15 minutes at 37°C. Cell suspension was then transferred to microtubes and immediately analyzed by flow cytometry. Loss of ™ψm was measured in the right lower quadrant.

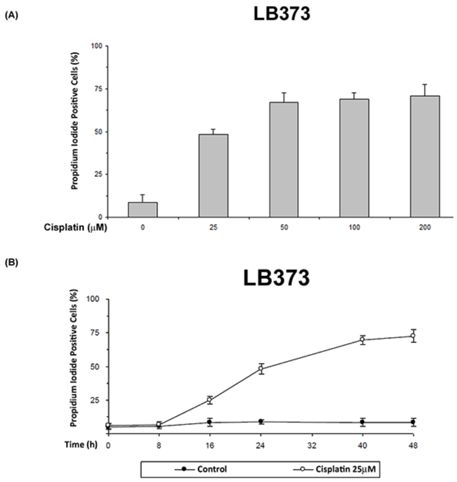

Supplementary Figure 1: Concentration and time response curve for cisplatin on LB373 melanoma cells. **(A)** LB373 melanoma cells($2x10^5$) were treated for 24h with different concentrations of cisplatin. Cell death was monitored by the loss of propidium iodide exclusion. **(B)** Cells ($2x10^5$) were treated with 25 µM of cisplatin for different times. Cell death was monitored by loss of propidium iodide exclusion. EC50 value for cisplatin treatment of the LB373 cell line was 25 µM for 24 h treatment.

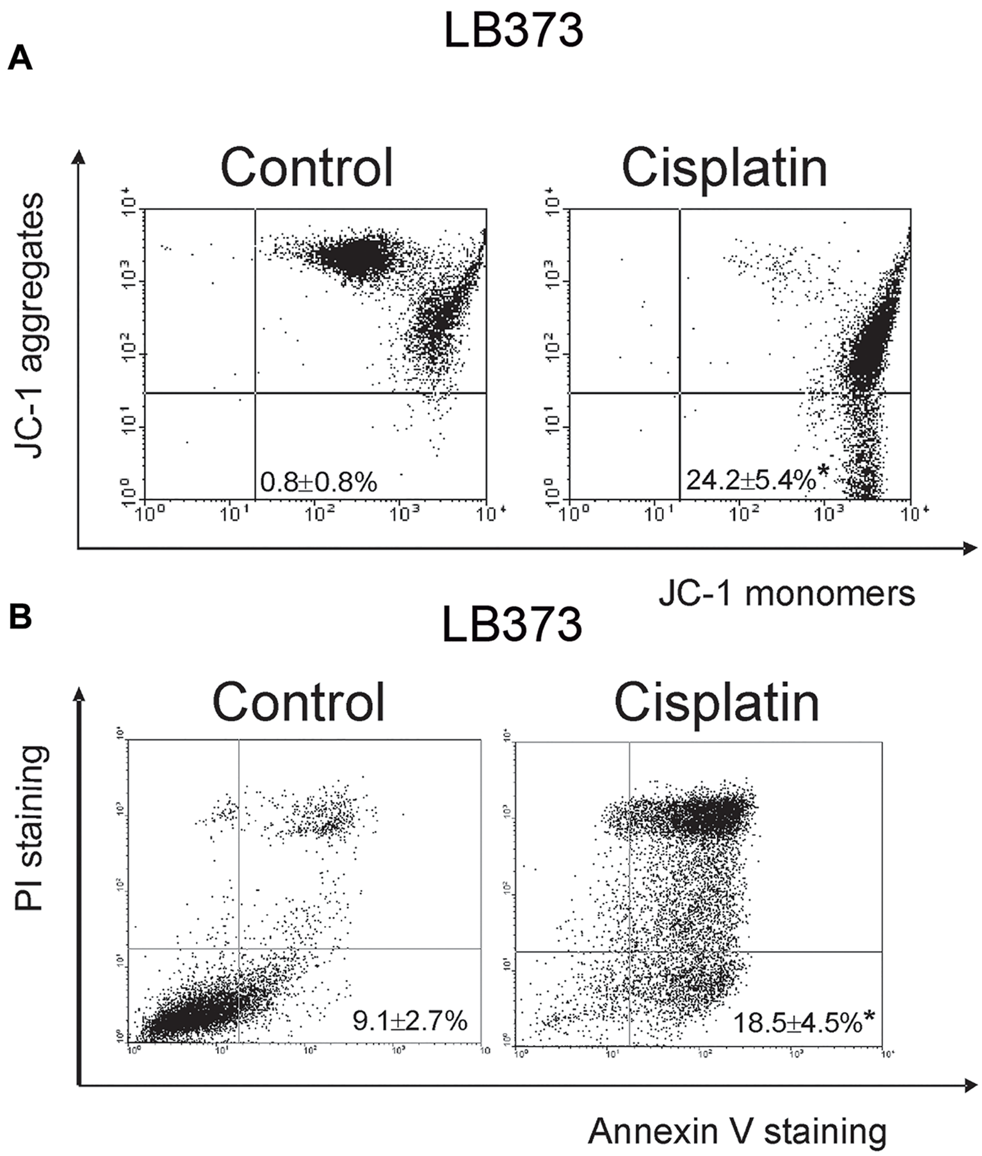

Supplementary Figure 2: Cisplatin modifies mitochondrial membrane potential and induces apoptosis in LB373 cells. **(A)** Cells were incubated with 200nM of JC-1 for 15 minutes and JC-1 monomers and aggregates were measured by flow cytometry. Loss of JC-1 aggregates was quantified in the graphs. **(B)** Cells were incubated with Annexin V FITC conjugate for 15 minutes and with propidium iodide (PI) for 5 minutes. Annexin V and PI staining were evaluated by flow cytometry. Percent Annexin V +/PI- cells is shown in the graphs (*$p<0.01$). Cells were treated with 25 µM cisplatin for 24 h. Representative data of at least three independent assays are shown.

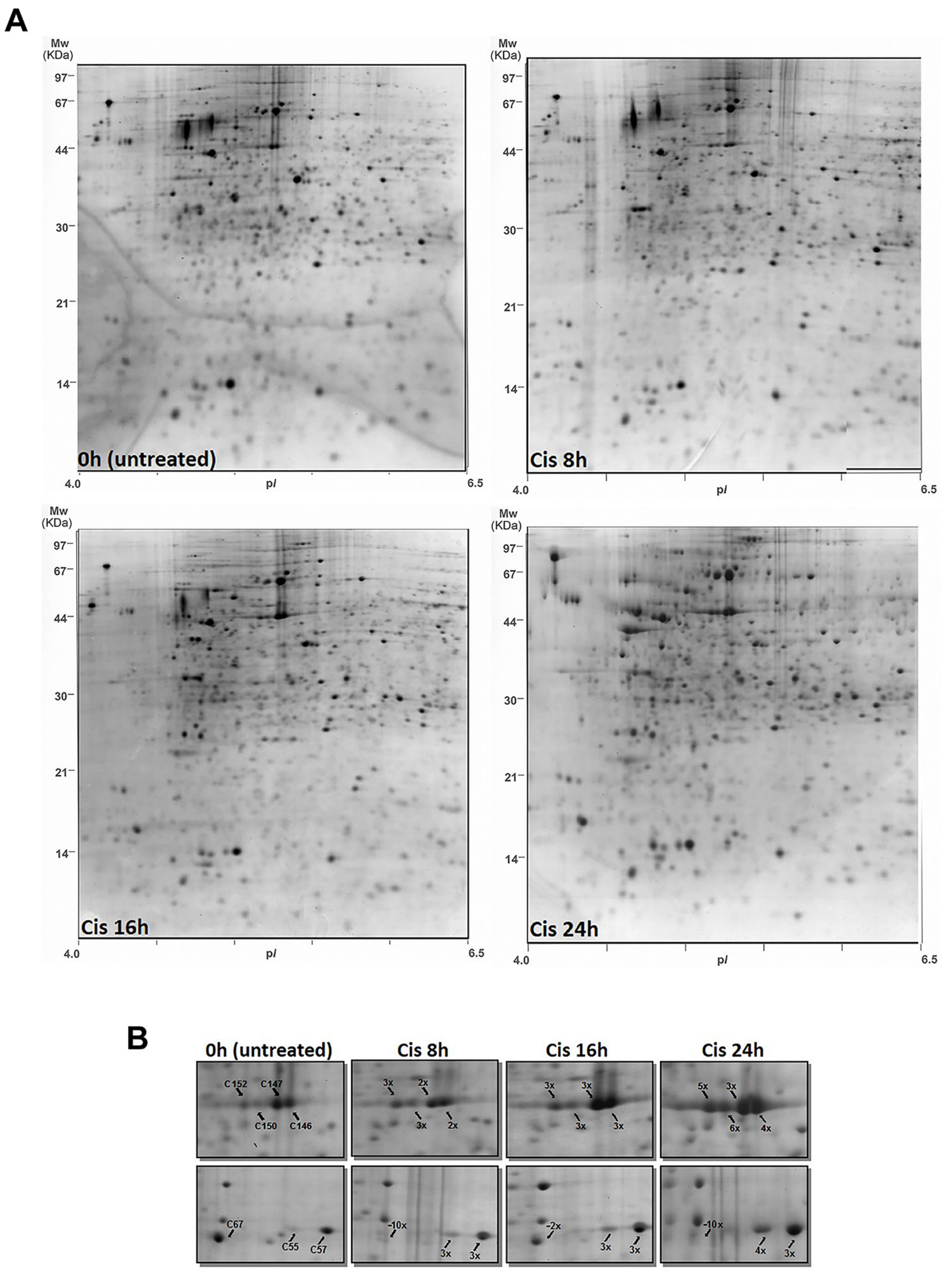

Supplementary Figure 3: IEF-SDS-PAGE analysis of cisplatin-treated melanoma cells. LB373 cells were treated and protein extracts from treated cells and control conditions were separated by IEF-SDS-PAGE as described in Materials and Methods. **(A)** 2D-Maps of untreated and 25 µM cisplatin-treated cells. **(B)** Marked spots show examples of differential protein accumulation upon cisplatin treatment. Values indicate intensity variation compared to untreated cells.

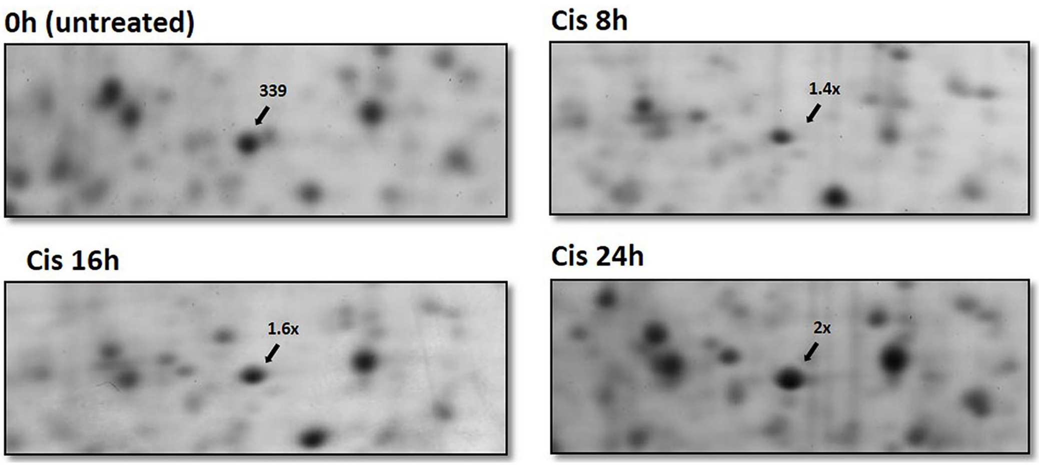

Supplementary Figure 4: Prohibitin accumulation in melanoma cells after cisplatin treatment. LB373 cells were treated and their extracts separated by IEF-SDS-PAGE as described in Materials and Methods. Prohibitin (spot 339). Values indicate intensity variation compared to untreated cells.

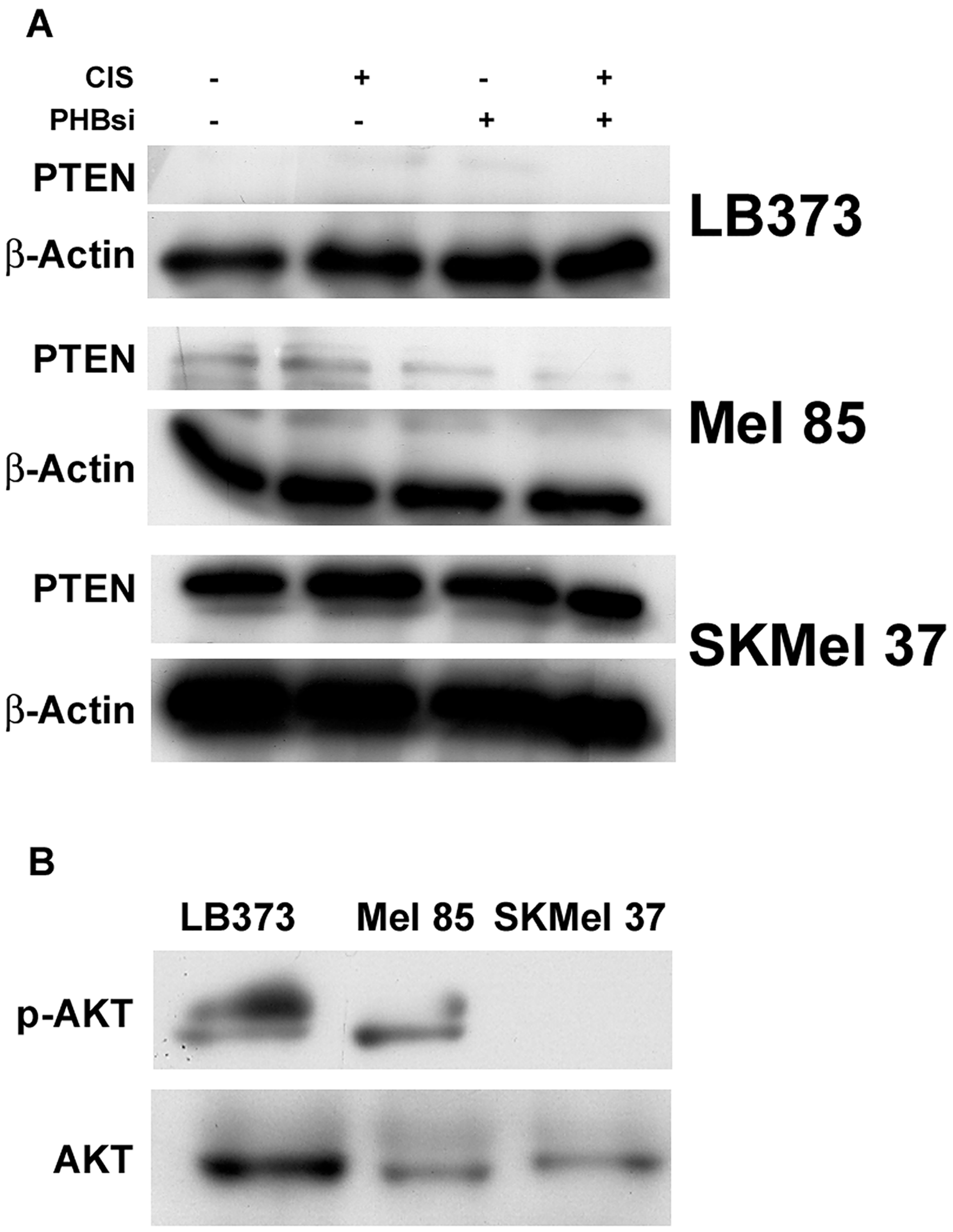

Supplementary Figure 5: PTEN (A) and phospho-AKT (B) expression in LB373, Mel 85 and SKMel 37 melanoma cells. Cells were treated as described in Materials and Methods. **(A)** LB373 and Mel 85 melanoma cells do not express PTEN, even under cisplatin treatment or PHB inhibition by siRNA. SKMel 37 cells express high levels of PTEN. Cisplatin treatment and inhibition of PHB by siRNA do not affect PTEN levels in SKMel 37 cell line **(B)** LB373 and Mel 85 cells have high levels of AKT phosphorylated as they do not express PTEN. As SKMel 37 cells express high levels of active PTEN, this cell line does not have phosphorylation in AKT. Data are representative of two independent assays.

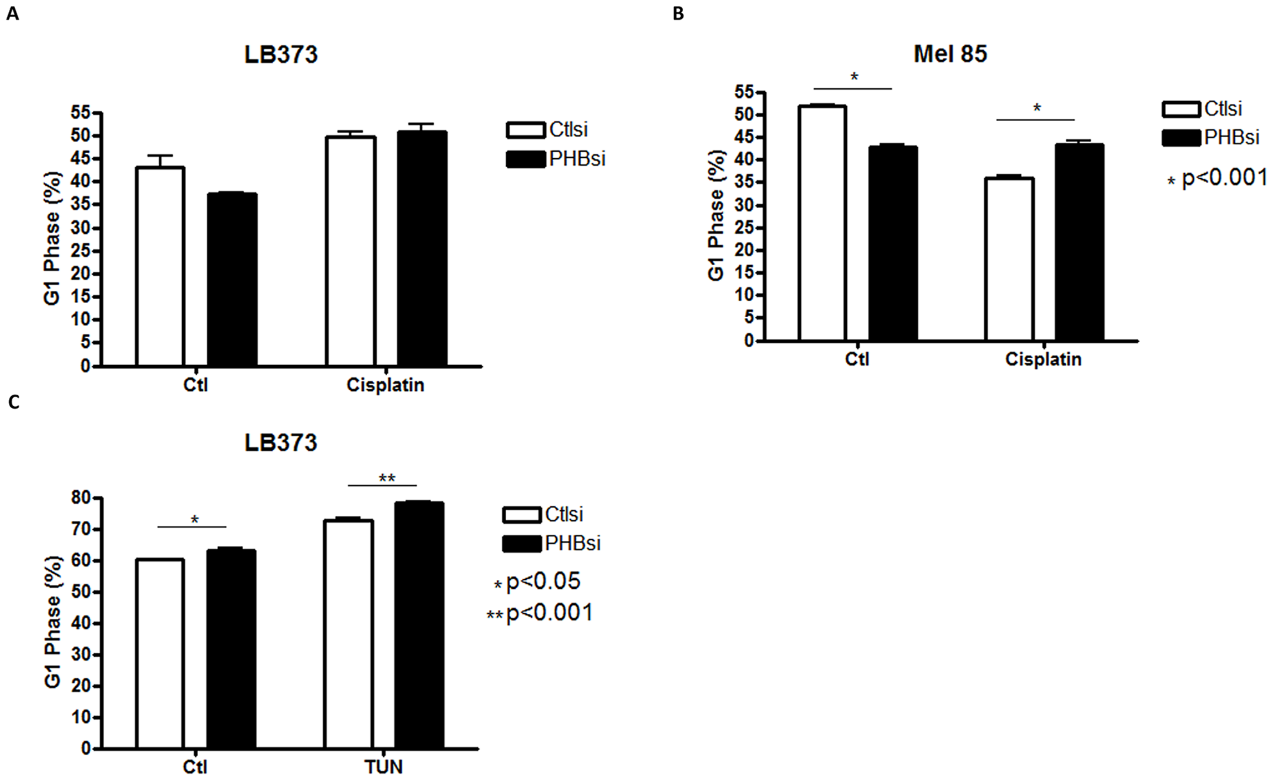

Supplementary Figure 6: G1 phase profile of LB373 and Mel 85 melanoma cells after cisplatin and tunicamycin treatment. Cell treatment and siRNA assay was performed as described in materials and methods. G1 phase DNA content was analyzed using propidium iodide as described in materials and methods. **(A)** LB373 cells had no difference in G1 phase after cisplatin treatment, even after PHB inhibition. **(B)** In Mel 85 cells, PHB inhibition lead to an inhibition in G1 phase of the cell cycle. Under cisplatin treatment, PHB inhibition increases the number of cells in G1 phase. **(C)** Tunicamycin treatment increases the G1 phase in LB373 cells. This increase is higher after PHB inhibition.
